# Pet Ownership, Living Alone, and Cognitive Decline Among Adults 50 Years and Older

**DOI:** 10.1001/jamanetworkopen.2023.49241

**Published:** 2023-12-26

**Authors:** Yanzhi Li, Wanxin Wang, Liwan Zhu, Liwen Yang, Herui Wu, Xiaojuan Zhang, Lan Guo, Ciyong Lu

**Affiliations:** 1 Department of Medical Statistics and Epidemiology, School of Public Health, Sun Yat-sen University, Guangzhou, China

## Abstract

**Importance:**

It remains unclear whether pet ownership is associated with cognitive decline and to what extent pet ownership mitigates the association between living alone and cognitive decline.

**Objective:**

To explore the association of pet ownership with cognitive decline, the interaction between pet ownership and living alone, and the extent to which pet ownership mitigates the association between living alone and cognitive decline in older adults.

**Design, Setting, and Participants:**

This cohort study used data from waves 5 (June 2010 to July 2011) to 9 (from June 2018 to July 2019) in the English Longitudinal Study of Ageing. Participants included adults 50 years and older. Data were analyzed from April 1 to June 30, 2023.

**Exposures:**

Pet ownership and living alone in wave 5.

**Main Outcomes and Measures:**

In waves 5 to 9, verbal memory and verbal fluency were assessed, and composite verbal cognition was further calculated.

**Results:**

Of the 7945 participants included, the mean (SD) age was 66.3 (8.8) years, and 4446 (56.0%) were women. Pet ownership was associated with slower rates of decline in composite verbal cognition (β = 0.008 [95% CI, 0.002-0.014] SD/y), verbal memory (β = 0.006 [95% CI, 0.001-0.012] SD/y), and verbal fluency (β = 0.007 [95% CI, 0.001-0.013] SD/y). Three-way interaction tests showed that living alone was a significant modifier in all 3 associations. Stratified analyses showed that pet ownership was associated with slower rates of decline in composite verbal cognition (β = 0.023 [95% CI, 0.011-0.035] SD/y), verbal memory (β = 0.021 [95% CI, 0.008-0.034] SD/y), and verbal fluency (β = 0.018 [95% CI, 0.005-0.030] SD/y) among individuals living alone, but not among those living with others. Joint association analyses showed no significant difference in rates of decline in composite verbal cognition, verbal memory, or verbal fluency between pet owners living alone and pet owners living with others.

**Conclusions and Relevance:**

In this cohort study, pet ownership was associated with slower rates of decline in verbal memory and verbal fluency among older adults living alone, but not among those living with others, and pet ownership offset the associations between living alone and declining rates in verbal memory and verbal fluency. Further studies are needed to assess whether pet ownership slows the rate of cognitive decline in older adults living alone.

## Key Points

**Questions:**

Is pet ownership associated with cognitive decline in older adults, and how does pet ownership mitigate the association between living alone and the rate of cognitive decline?

**Findings:**

In this cohort study of 7945 participants 50 years and older, pet ownership was associated with slower rates of decline in verbal memory and verbal fluency among individuals living alone, but not among those living with others. Pet ownership offset the association between living alone and declining rates of verbal memory and verbal fluency.

**Meaning:**

These findings suggest that pet ownership may be associated with slower cognitive decline among older adults living alone.

## Teaser

This cohort study explores the association between pet ownership and rates of cognitive decline, the interaction between pet ownership and living alone, and the extent to which pet ownership mitigates the association between living alone and cognitive decline in older adults.

## Introduction

Older adults tend to experience cognitive decline.^1,2^ As the population ages and life expectancy increases, a major public health issue is the deterioration of cognitive function in older adults.^3,4^ It is estimated that the number of people with dementia worldwide will increase from 57 million in 2019 to 153 million in 2050.^4^ The deterioration of cognitive function not only seriously impairs individuals’ well-being but also brings a huge burden to their caregivers, as well as the financial and health systems of society.^5,6^ No effective therapy is currently available to successfully reverse cognitive decline or treat dementia.^6^ Thus, identifying high-risk populations and modifiable risk factors is crucial for formulating public health interventions and promoting healthy aging.

In the past few decades, the proportion of individuals living alone has shown an upward trend. In 2021, the proportion of single-person households in the United Kingdom (UK) and the US reached 29.4% and 28.5%, respectively.^7,8^ A recent meta-analysis of 12 studies^9^ reported that older adults living alone are at high risk for developing dementia and that the population-attributable fraction for living alone is 8.9%. This figure will increase given that the proportion of older adults living alone is on the rise. Currently, it is critical to identify modifiable factors that reduce dementia risk in older adults living alone.

Loneliness is a potential mediator in the association of living alone with dementia among older adults.^10,11,12^ Contrary to living alone,^10^ pet ownership (eg, raising dogs and cats) is related to reduced loneliness,^13,14,15^ an important risk factor for dementia and cognitive decline.^11,12,16^ However, the association between pet ownership and the rate of cognitive decline has not been fully explored, and the existing findings remain controversial.^17,18,19,20,21^ Several cross-sectional studies have found that pet ownership is associated with better verbal memory,^17^ story memory,^17^ executive function (ie, serial sevens subtraction and clock-drawing tests),^18,19^ processing speed,^20^ and orientation function.^20^ However, some cross-sectional studies^19,21^ have reported that pet ownership is not associated with verbal memory or executive function (ie, backward number counting tests). To date, prospective longitudinal studies to elucidate the association between pet ownership and the rate of cognitive decline are lacking. Moreover, whether there is an interaction between pet ownership and living alone and to what extent pet ownership mitigates the association between living alone and the rate of cognitive decline is unclear. Therefore, this cohort study aimed (1) to explore the association between pet ownership and the rate of cognitive decline; (2) to evaluate the interaction between pet ownership and living alone; and (3) to assess to what extent pet ownership mitigates the association between living alone and the rate of cognitive decline in older adults.

## Methods

### Study Design and Participants

In this cohort study, data were obtained from the English Longitudinal Study of Ageing (ELSA), an ongoing, prospective, and nationally representative cohort of community-dwelling adults 50 years or older in the UK that has been previously described in detail.^22^ Briefly, the ELSA began collecting data in wave 1 (March 2002 March 2003), and participants were biennially followed up through wave 9 (June 2018 to July 2019). The ELSA data sets were available from the UK Data Service.^23^ The ELSA received ethical approval from the London Multicenter Research Ethics Committee and complied with the Declaration of Helsinki.^24^ All participants signed the informed consent document. This study followed the Strengthening the Reporting of Observational Studies in Epidemiology (STROBE) reporting guideline.

Information on pet ownership was investigated in wave 5, so wave 5 was used as the baseline for this study. We used data from wave 5 on pet ownership, living alone, and potential covariates, as well as data from waves 5 to 9 on cognitive function (eFigure 1 in Supplement 1). Wave 5 was conducted from June 2010 to July 2011; wave 6, from May 2012 to June 2013; wave 7, from June 2014 to May 2015; wave 8, from May 2016 to June 2017; and wave 9, from June 2018 to July 2019. A total of 10 095 individuals 50 years and older participated in wave 5. Based on inclusion and exclusion criteria, 7945 participants were eligible for the main analyses. The detailed selection process is shown in eFigure 2 in Supplement 1.

### Assessment of Pet Ownership

In wave 5, information on pet ownership was collected by asking participants: “Do you keep any household pets inside your house/flat?”^21^ Options included yes and no.

### Assessment of Living Alone

In wave 5, living arrangements were investigated based on the number of residents recorded in each household. If only 1 household member (ie, the participant) was recorded in the household, they were defined as living alone. Otherwise, they were defined as living with others.^25^

### Assessment of Cognitive Function

In waves 5 to 9, verbal memory and verbal fluency were evaluated. Detailed measurements are provided in the eMethods in Supplement 1. There were significant differences in the means and SDs of verbal memory and verbal fluency scores.^26,27,28^ Referring to previous studies,^26,27,28^ to allow for direct comparisons across different domains and generate a composite verbal cognition score, we calculated a standardized *z* score. First, we calculated the *z* score for each domain by subtracting the mean and dividing it by the SD in wave 5. Then, we calculated the mean of the 2 *z* scores and used the same approach to obtain the *z* score of composite verbal cognition. A cognitive *z* score of −1.00 at any wave represents 1 SD below the mean cognitive score in wave 5.

### Assessment of Potential Covariates

All potential covariates were assessed in wave 5, including age, sex (men or women), self-reported race and ethnicity (White compared with other races or ethnicities [including Asian, Asian British, Black, Black British, multiethnic, and other]), educational level (high, middle, or low), employment status (employed, unemployed, or retired), wealth, social isolation score, smoking status (current or noncurrent [includes former and never]), alcohol consumption status, physical activity, self-rated general health (excellent, very good, good, fair, or poor), depressive symptoms (yes or no), self-reported diabetes (yes or no), self-reported hypertension (yes or no), and self-reported cardiovascular disease (yes or no). The detailed evaluation and classification methods for potential covariates are provided in the eMethods in Supplement 1.

### Statistical Analyses

Data were analyzed from April 1 to June 30, 2023. Participants’ characteristics in wave 5 were summarized according to pet ownership (yes or no). Data were shown as mean (SD) for continuous variables or frequency (percentage) for categorical variables and were compared using Pearson χ^2^ tests or independent 2-sample *t* tests, as appropriate.

First, we used a linear mixed model to explore the association between pet ownership and the rate of cognitive decline (in SD per year), with the intercept and slope of follow-up time fitted as random effects at the participant level. The follow-up time was calculated by subtracting the date of the cognitive function assessment in wave 5 from the dates of the cognitive function assessment in subsequent waves. Model 1 included pet ownership, time, pet ownership × time, age, sex, and race and ethnicity. Model 2 additionally included educational level, employment status, wealth quintiles, living alone, social isolation, smoking status, alcohol consumption, physical activity, self-rated general health, depressive symptoms, diabetes, hypertension, and cardiovascular disease. Results were presented as β coefficients and 95% CIs. We used the same method to evaluate the association between living alone and the rate of cognitive decline.

Second, we assessed the moderating role of living alone in the association between pet ownership and cognitive function by including pet ownership, living alone, time, pet ownership × time, living alone × time, pet ownership × living alone, pet ownership × living alone × time, and the covariates in model 2. Stratified analyses of living alone were further conducted if the 3-way interaction (ie, pet ownership × living alone × time) was statistically significant.^29^

Third, to assess whether pet ownership mitigates the association of living alone with cognitive decline, we investigated the joint associations of pet ownership and living alone with the rate of cognitive decline. Participants were classified into 4 groups according to living alone (yes or no) and pet ownership (yes or no), with the combination of not living alone and pet ownership as the reference. Linear mixed models included the combination of living alone and pet ownership, time, the combination of living alone and pet ownership × time, and the covariates in model 2.

Last, we conducted 2 sensitivity analyses. An inverse probability weighting analysis assessed whether the missing data affected the results.^30,31^ Detailed descriptions are provided in the eMethods in Supplement 1. In addition, because pet ownership was only investigated in wave 5 and living alone was investigated in waves 5 to 9, we explored the association between time-varying living alone and cognitive decline.

All statistical analyses used Stata, version 17.0 (StataCorp LLC). Statistical significance was defined as 2-tailed *P* < .05.

## Results

### Characteristics of Participants

We included 7945 participants with a mean (SD) age of 66.3 (8.8) years in wave 5, among whom 3499 (44.0%) were men and 4446 (56.0%) were women; 7746 (97.5%) were White and 199 (2.5%) were other race or ethnicity; 2791 (35.1%) owned pets; and 2139 (26.9%) lived alone (Table 1). The cumulative attrition rates were 2.0% for wave 6, 14.7% for wave 7, 24.0% for wave 8, and 32.7% for wave 9 (eFigure 1 in Supplement 1). There was no difference in baseline characteristics between participants included and those lost to follow-up except for hypertension and cardiovascular disease (eTable 1 in Supplement 1). The follow-up time (eTable 2 in Supplement 1) and rates of loss to follow-up (eTable 3 in Supplement 1) did not differ according to pet ownership, living alone, or the combination of pet ownership and living alone. Cumulative rates of loss to follow-up also did not differ according to pet ownership, living alone, or the combination of pet ownership and living alone (eFigure 3 in Supplement 1). Actual verbal memory and verbal fluency scores during waves 5 to 9 are presented in eFigure 4 in Supplement 1.

**Table 1.** Characteristics of Participants by Pet Ownership in Wave 5

Characteristic	Participant group^a^			*P* value^b^
	Total (N = 7945)	Pet ownership		
		Yes (n = 2791)	No (n = 5154)	
Age, mean (SD), y	66.3 (8.8)	63.8 (7.9)	67.6 (9.0)	<.001
Sex				
Men	3499 (44.0)	1210 (43.4)	2289 (44.4)	.36
Women	4446 (56.0)	1581 (56.6)	2865 (55.6)	
Race and ethnicity				
White	7746 (97.5)	2761 (98.9)	4985 (96.7)	<.001
Other^c^	199 (2.5)	30 (1.1)	169 (3.3)	
Educational level				
High	2549 (32.1)	869 (31.1)	1680 (32.6)	.02
Middle	638 (8.0)	256 (9.2)	382 (7.4)	
Low	4758 (59.9)	1666 (59.7)	3092 (60.0)	
Employment status				
Employed	2553 (32.1)	1145 (41.0)	1408 (27.3)	<.001
Unemployed	862 (10.8)	369 (13.2)	493 (9.6)	
Retired	4530 (57.0)	1277 (45.8)	3253 (63.1)	
Wealth quintiles				
1 (Poorest)	1117 (14.1)	427 (15.3)	690 (13.4)	<.001
2	1423 (17.9)	489 (17.5)	934 (18.1)	
3	1421 (17.9)	440 (15.8)	981 (19.0)	
4	1528 (19.2)	502 (18.0)	1026 (19.9)	
5 (Richest)	2456 (30.9)	933 (33.4)	1523 (29.5)	
Living alone	2139 (26.9)	619 (22.2)	1520 (29.5)	<.001
Social isolation score, mean (SD)^d^	0.87 (0.88)	0.79 (0.86)	0.92 (0.90)	<.001
Current smoking	961 (12.1)	445 (15.9)	516 (10.0)	<.001
Alcohol consumption				
Less than weekly	3139 (39.5)	1152 (41.3)	1987 (38.6)	<.001
1-4 d/wk	2926 (36.8)	966 (34.6)	1960 (38.0)	
5-7 d/wk	1880 (23.7)	673 (24.1)	1207 (23.4)	
Physical activity				
Light	1475 (18.6)	421 (15.1)	1054 (20.5)	<.001
Moderate	3543 (44.6)	1323 (47.4)	2220 (43.1)	
Vigorous	2927 (36.8)	1047 (37.5)	1880 (36.5)	
Self-rated general health				
Excellent	1030 (13.0)	381 (13.7)	649 (12.6)	.003
Very good	2482 (31.2)	827 (29.6)	1655 (32.1)	
Good	2557 (32.2)	876 (31.4)	1681 (32.6)	
Fair	1392 (17.5)	505 (18.1)	887 (17.2)	
Poor	484 (6.1)	202 (7.2)	282 (5.5)	
Depressive symptoms	930 (11.7)	393 (14.1)	537 (10.4)	<.001
Hypertension	3133 (39.4)	1026 (36.8)	2107 (40.9)	<.001
Diabetes	825 (10.4)	272 (9.7)	553 (10.7)	.17
Cardiovascular disease	1726 (21.7)	568 (20.4)	1158 (22.5)	.03
Verbal memory score, mean (SD)^e^	10.8 (3.5)	11.1 (3.3)	10.6 (3.6)	<.001
Verbal fluency score, mean (SD)^f^	21.4 (6.6)	22.0 (6.6)	21.0 (6.6)	<.001
Verbal memory *z* score, mean (SD)^g^	0.00 (1.00)	0.09 (0.95)	−0.05 (1.02)	<.001
Verbal fluency *z* score, mean (SD)^g^	0.00 (1.00)	0.10 (1.00)	−0.06 (1.00)	<.001
Composite verbal cognition *z* score, mean (SD)^h^	0.00 (1.00)	0.11 (0.95)	−0.06 (1.02)	<.001

^a^ Unless otherwise indicated, data are expressed as No. (%) of participants. Percentages have been rounded and may not total 100.

^b^ Independent 2-sample *t* tests were used to compare the means of continuous variables. Pearson χ^2^ tests were performed to compare the distribution of categorical variables.

^c^ Includes Asian, Asian British, Black, Black British, multiethnic, and other.

^d^ Scores range from 0 to 5, with higher scores indicating greater isolation.

^e^ Scores range from 0 to 20, with higher scores indicating better cognitive performance in verbal memory.

^f^ Scores are calculated as a list of animal names given within 1 minute, with no upper limits. Higher scores indicate better cognitive performance in verbal fluency.

^g^ Calculated as the *z* score for each domain by subtracting the mean and dividing it by the SD in wave 5. A cognitive *z* score of −1.00 at any wave represents 1 SD below the mean cognitive score in wave 5.

^h^ Calculated as the mean of the 2 *z* scores and subtracting the mean and dividing it by the SD in wave 5.

### Individual Associations of Pet Ownership and Living Alone With Cognitive Decline

**Figure 1.**
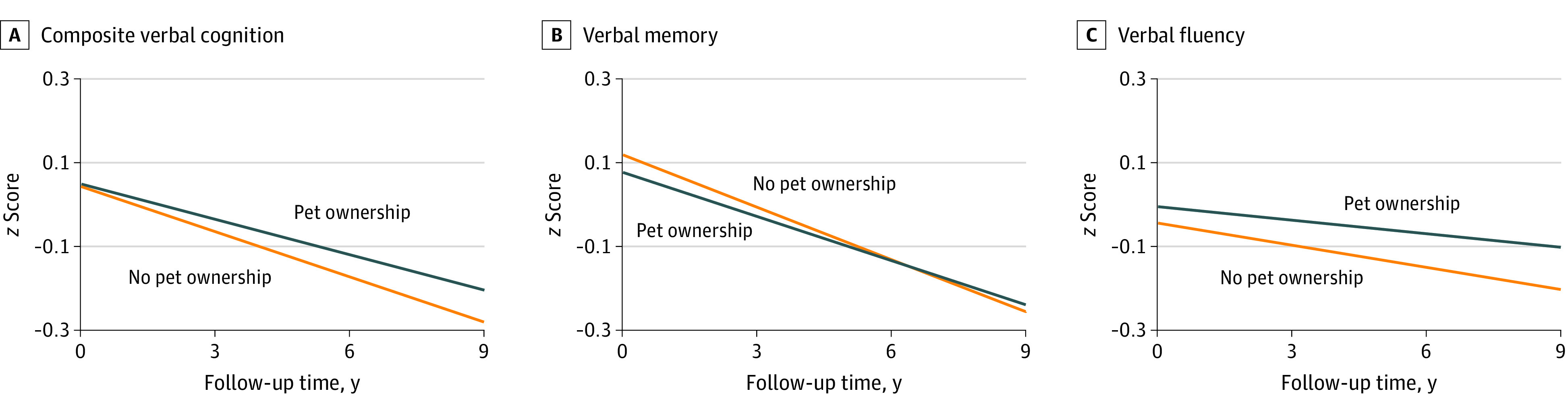
Estimated Cognition *z* Scores During Waves 5 to 9 by Pet Ownership in Wave 5 Estimated *z* scores were calculated in SD units. Covariates were set to the following values: 65 years of age, female, White race, high educational level, retired, the third quintile of wealth, living alone, social isolation score of 1, currently nonsmoking, alcohol consumption less than once a week, moderate physical activity, good self-rated general health, and no depressive symptoms, hypertension, diabetes, or cardiovascular disease.

After adjusting for potential covariates (Table 2, model 2), compared with nonowners, pet owners had a slower rate of decline in composite verbal cognition (β coefficient, 0.008 [95% CI, 0.002-0.014] SD/y), verbal memory (β coefficient, 0.006 [95% CI, 0.001-0.012] SD/y), and verbal fluency (β coefficient, 0.007 [95% CI, 0.001-0.013] SD/y). Figure 1 shows estimated cognition *z* scores during waves 5 to 9 by pet ownership. In contrast, compared with individuals living with others (Table 2, model 2), those living alone showed a faster decline in composite verbal cognition (β coefficient, −0.021 [95% CI, −0.027 to −0.014] SD/y), verbal memory (β coefficient, −0.018 [95% CI, −0.025 to −0.011] SD/y), and verbal fluency (β coefficient, −0.015 [95% CI, −0.022 to −0.008] SD/y).

**Table 2.** Individual Associations of Pet Ownership and Living Alone in Wave 5 With Cognitive Decline During Waves 5 to 9

Variable	No. of participants	Model 1^a^		Model 2^b^	
		β Coefficient (95% CI)	*P* value	β Coefficient (95% CI)	*P* value
**Pet ownership**					
Composite verbal cognition					
No pet ownership × time	5154	0 [Reference]		0 [Reference]	
Pet ownership × time	2791	0.008 (0.002 to 0.014)	.009	0.008 (0.002 to 0.014)	.009
Verbal memory					
No pet ownership × time	5154	0 [Reference]		0 [Reference]	
Pet ownership × time	2791	0.006 (0.001 to 0.012)	.04	0.006 (0.001 to 0.012)	.04
Verbal fluency					
No pet ownership × time	5154	0 [Reference]		0 [Reference]	
Pet ownership × time	2791	0.007 (0.001 to 0.013)	.03	0.007 (0.001 to 0.013)	.03
**Living alone**					
Composite verbal cognition					
Not living alone × time	5806	0 [Reference]		0 [Reference]	
Living alone × time	2139	−0.021 (−0.027 to −0.014)	<.001	−0.021 (−0.027 to −0.014)	<.001
Verbal memory					
Not living alone × time	5806	0 [Reference]		0 [Reference]	
Living alone × time	2139	−0.018 (−0.025 to −0.012)	<.001	−0.018 (−0.025 to −0.011)	<.001
Verbal fluency					
Not living alone × time	5806	0 [Reference]		0 [Reference]	
Living alone × time	2139	−0.015 (−0.022 to −0.008)	<.001	−0.015 (−0.022 to −0.008)	<.001

^a^ For pet ownership, the model included pet ownership, time, pet ownership × time, and covariates (ie, age, sex, race and ethnicity in wave 5). For living alone, the model included living alone, time, living alone × time, and covariates (ie, age, sex, and race and ethnicity in wave 5).

^b^ For pet ownership, the model included pet ownership, time, pet ownership × time, and covariates (ie, age, sex, race and ethnicity, educational level, employment status, wealth, smoking status, alcohol consumption, physical activity, social isolation score, self-rated general health, depressive symptoms, hypertension, diabetes, cardiovascular disease, and living alone in wave 5). For living alone, the model included living alone, time, living alone × time, and covariates (ie, age, sex, race and ethnicity, educational level, employment status, wealth, smoking status, alcohol consumption, physical activity, social isolation score, self-rated general health, depressive symptoms, hypertension, diabetes, cardiovascular disease, and pet ownership in wave 5).

### Moderating Role of Living Alone

**Figure 2.**
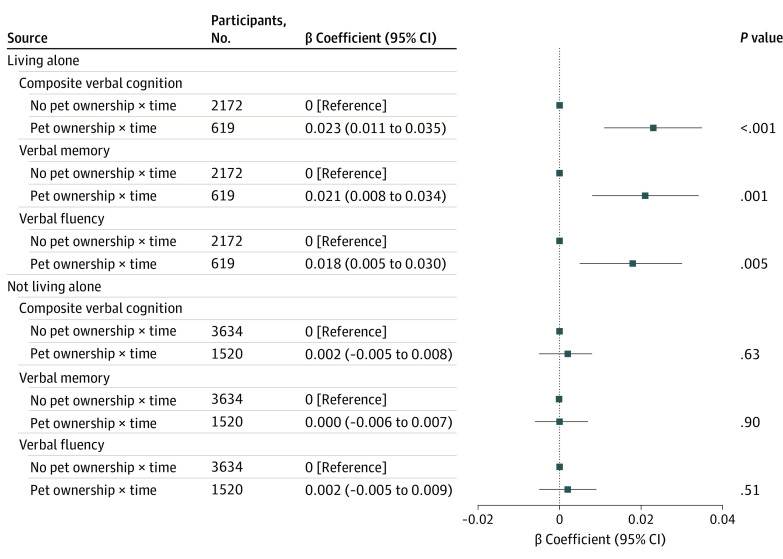
Associations of Pet Ownership in Wave 5 With Cognitive Decline During Waves 5 to 9, Stratified by Living Alone in Wave 5 The model included pet ownership, time, pet ownership × time, and covariates (ie, age, sex, race and ethnicity, educational level, employment status, wealth, smoking status, alcohol consumption, physical activity, social isolation score, self-rated general health, depressive symptoms, hypertension, diabetes, and cardiovascular disease in wave 5).

As shown in eTable 4 in Supplement 1, living alone was a significant modifier in the associations of pet ownership with rates of decline in the composite verbal cognition (β coefficient, 0.021 [95% CI, 0.007-0.035] SD/y for 3-way interaction), verbal memory (β coefficient, 0.020 [95% CI, 0.006-0.035] SD/y for 3-way interaction), and verbal fluency (β coefficient, 0.015 [95% CI, 0.001-0.030] SD/y for 3-way interaction). Stratified analyses showed that pet owners had slower rates of decline in composite verbal cognition (β coefficient, 0.023 [95% CI, 0.011-0.035] SD/y), verbal memory (β coefficient, 0.021 [95% CI, 0.008-0.034] SD/y), and verbal fluency (β coefficient, 0.018 [95% CI, 0.005-0.030] SD/y) among individuals living alone, but not among those living with others (Figure 2). eFigure 5 in Supplement 1 presents estimated cognition *z* scores during waves 5 to 9 by pet ownership, stratified by living alone.

### Joint Associations of Pet Ownership and Living Alone With Cognitive Decline

**Figure 3.**
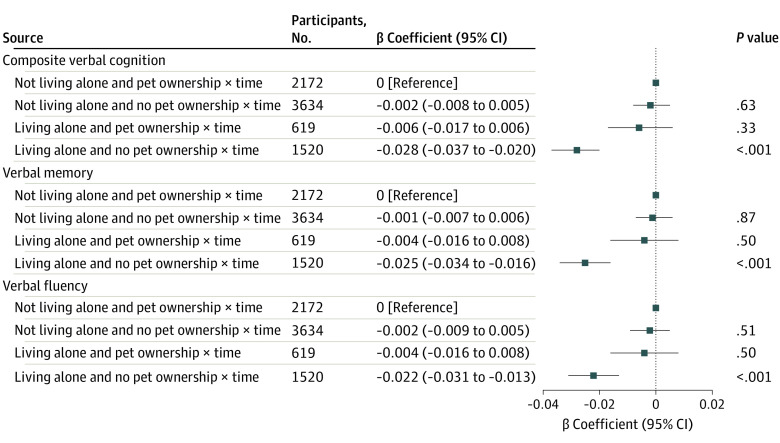
Joint Associations of Pet Ownership and Living Alone in Wave 5 With Cognitive Decline During Waves 5 to 9 The model included the combination of living alone and pet ownership, time, the combination of living alone and pet ownership × time, and covariates (ie, age, sex, race and ethnicity, educational level, employment status, wealth, smoking status, alcohol consumption, physical activity, social isolation score, self-rated general health, depressive symptoms, hypertension, diabetes, and cardiovascular disease in wave 5).

Compared with pet owners living with others (Figure 3), nonowners living alone had faster rates of decline in composite verbal cognition (β coefficient, −0.028 [95% CI, −0.037 to −0.020] SD/y), verbal memory (β coefficient, −0.025 [95% CI, −0.034 to −0.016] SD/y), and verbal fluency (β coefficient, −0.022 [95% CI, −0.031 to −0.013] SD/y), but nonowners living with others or pet owners living alone did not. eFigure 6 in Supplement 1 displays estimated cognition *z* scores during waves 5 to 9 by pet ownership and living alone.

### Sensitivity Analyses

The findings from the inverse probability weighting analysis showed no meaningful differences from those of the primary analysis (eTables 5-9 in Supplement 1). In addition, the association between time-varying living alone and cognitive decline was similar to that in the main analyses (eTable 10 in Supplement 1).

## Discussion

To the best of our knowledge, limited evidence is available on the association between pet ownership and rate of cognitive decline, as well as whether pet ownership mitigates the association between living alone and the rate of cognitive decline in older adults. Using nationally representative data from the ELSA, this prospective cohort study found that pet ownership was associated with slower rates of decline in verbal memory, verbal fluency, and composite verbal cognition among older adults living alone, but not among those living with others. Moreover, pet ownership completely offset the associations of living alone with declining rates in verbal memory, verbal fluency, and composite verbal cognition. Our findings provide innovative insights for developing public health policies to slow cognitive decline in older adults living alone.

Consistent with our findings, a previous cross-sectional study^17^ has shown that pet ownership is associated with better verbal memory. Other cross-sectional studies^18,19^ have used the performance in serial sevens subtraction and clock-drawing tests to reflect executive function. Although the evaluation methods for executive function in those studies are inconsistent with ours (ie, verbal fluency test findings), they have found that pet ownership is associated with better executive function, which is similar to our findings.^18,19^ In a small-sample study (n = 637),^32^ participants recalled pet ownership during the past 10 years, and the investigators explored the association of pet ownership with cognitive decline from 10 years ago to the following 3 years. Friedmann et al^32^ found that after adjusting for age and comorbidities, the deterioration in verbal memory and executive function (ie, digit span tests) was slower for pet owners than nonowners, which is similar to our results. Although uncertain temporal relationships between pet ownership and cognitive decline, residual confounding, and recall bias may affect their analysis, their results support our findings to some extent.^32^ In contrast, previous cross-sectional studies^19,21^ did not find an association of pet ownership with verbal memory or executive function (ie, backward number counting tests). The reason for the inconsistent results might be differences in study design (eg, longitudinal vs cross-sectional) and evaluation instruments (eg, verbal fluency tests vs backward number counting tests). In addition, our prospective cohort study found that living alone moderated the association between pet ownership and rates of decline in verbal memory and verbal fluency. Stratified analyses showed that pet ownership was associated with a slower rate of decline in verbal memory and verbal fluency among older adults living alone, but not among adults living with others. Our results provide stronger evidence and more nuanced insights into the benefits of pet ownership on verbal memory and verbal fluency among older adults living alone. However, in addition to verbal memory and executive function, cognitive function also includes attention, reasoning, processing speed, accuracy, and so on. Another cross-sectional study^20^ has reported that pet ownership is associated with better processing speed and orientation. Therefore, a comprehensive cognition assessment is needed to explore the longitudinal correlation between pet ownership and global cognitive function.

Older adults living alone are at high risk for developing dementia,^9^ and living alone is a state that is not easily changed. It is worth noting that compared with pet owners living with others, pet owners living alone did not show faster rates of decline in verbal memory or verbal fluency. These findings preliminarily suggest that pet ownership might completely offset the association of living alone with faster rates of decline in verbal memory and verbal fluency among older adults. The population-attributable fraction of living alone for dementia was 8.9%,^9^ and the figure will increase as the proportion of people living alone increases.^7,8^ In addition, pet ownership constitutes a simple change. Therefore, randomized clinical trials should explore whether pet ownership can slow the rate of cognitive decline, especially in older adults living alone. If randomized clinical trials confirm our findings, pet ownership may help in slowing cognitive decline and preventing dementia.

### Limitations

This study has several limitations. First, cognitive function includes multiple dimensions (eg, episodic memory, executive function, attention, reasoning, processing speed, and accuracy), but this study only assessed verbal memory and verbal fluency, which represent a single aspect each of episodic memory and executive function, respectively. A comprehensive cognitive function assessment is needed to explore the association between pet ownership and global cognitive decline. Second, since information on pet ownership was only investigated in wave 5, we assumed that pet ownership remained constant from wave 5. However, pet ownership might vary with time, so it is necessary to validate our findings using time-varying pet ownership. Third, since the ELSA did not investigate the duration of pet ownership, we did not assess the association between the duration of pet ownership and cognitive function. Fourth, almost all the participants in the ELSA were White, so our findings are not generalizable to other racial and ethnic groups. Last, due to the nature of observational studies, the effect of unmeasured confounding factors (eg, *APOE* genotype) on our results cannot be eliminated, hindering the determination of a causal association.

## Conclusions

In this prospective cohort study, pet ownership was associated with slower rates of decline in verbal memory, verbal fluency, and composite verbal cognition among older adults living alone, but not among those living with others, and pet ownership completely offset the associations between living alone and decline in verbal memory, verbal fluency, and composite verbal cognition. These findings suggest that pet ownership might be beneficial for verbal memory and verbal fluency among older adults living alone. Randomized clinical trials are needed to assess whether pet ownership slows the rate of cognitive decline in older adults living alone.

## Notes

Supplement 1. **eMethods.** Assessment of Cognitive Function and Potential Covariates and Inverse Probability Weighting **eTable 1.** Baseline Characteristics of Participants Included in the Analysis and Participants Lost to Follow-Up **eTable 2.** Follow-Up Time of Participants During Waves 5 to 9 **eTable 3.** Rates of Loss to Follow-Up During Waves 5 to 9 **eTable 4.** Modifying Role of Living Alone in Wave 5 in the Association of Pet Ownership in Wave 5 With Cognitive Decline During Waves 5 to 9 **eTable 5.** The Inverse Probability Weighting Analysis: Association of Pet Ownership in Wave 5 With Cognitive Decline During Waves 5 to 9 **eTable 6.** The Inverse Probability Weighting Analysis: Association of Living Alone in Wave 5 With Cognitive Decline During Waves 5 to 9 **eTable 7.** The Inverse Probability Weighting Analysis: Modifying Role of Living Alone in Wave 5 in the Association of Pet Ownership in Wave 5 With Cognitive Decline During Waves 5 to 9 **eTable 8.** The Inverse Probability Weighting Analysis: Association of Pet Ownership in Wave 5 With Cognitive Decline During Waves 5 to 9, Stratified by Living Alone in Wave 5 **eTable 9.** The Inverse Probability Weighting Analysis: Joint Associations of Pet Ownership and Living Alone in Wave 5 With Cognitive Decline During Waves 5 to 9 **eTable 10.** Association of Time-Varying Living Alone With Cognitive Decline During Waves 5 to 9 **eFigure 1.** The Timeline and Cumulative Attrition Rates of This Study **eFigure 2.** The Inclusion and Exclusion Process of Participants in This Study **eFigure 3.** Cumulative Rates of Loss to Follow-Up During Waves 5 to 9 **eFigure 4.** Actual Verbal Memory and Verbal Fluency Scores During Waves 5 to 9 **eFigure 5.** Estimated Cognition *z* Scores (SD Units) During Waves 5 to 9 by Pet Ownership in Wave 5, Stratified by Living Alone in Wave 5 **eFigure 6.** Estimated Cognition *z* Scores (SD Units) During Waves 5 to 9 by the Combination of Pet Ownership and Living Alone in Wave 5
eReferences

Supplement 2.
Data Sharing Statement
