# Supplementary material for: Pet Ownership, Living Alone, and Cognitive Decline Among Adults 50 Years and Older
Source: JAMA Netw Open. 2023 Dec 26;6(12):e2349241. doi: 10.1001/jamanetworkopen.2023.49241 (PMC10751597; doi:10.1001/jamanetworkopen.2023.49241)
Supplement: Supplement 1. — eMethods. Assessment of Cognitive Function and Potential Covariates and Inverse Probability Weighting eTable 1. Baseline Characteristics of Participants Included in the Analysis and Participants Lost to Follow-Up eTable 2. Follow-Up Time of Participants During Waves 5 to 9 eTable 3. Rates of Loss to Follow-Up During Waves 5 to 9 eTable 4. Modifying Role of Living Alone in Wave 5 in the Association of Pet Ownership in Wave 5 With Cognitive Decline During Waves 5 to 9 eTable 5. The Inverse Probability Weighting Analysis: Association of Pet Ownership in Wave 5 With Cognitive Decline During Waves 5 to 9 eTable 6. The Inverse Probability Weighting Analysis: Association of Living Alone in Wave 5 With Cognitive Decline During Waves 5 to 9 eTable 7. The Inverse Probability Weighting Analysis: Modifying Role of Living Alone in Wave 5 in the Association of Pet Ownership in Wave 5 With Cognitive Decline During Waves 5 to 9 eTable 8. The Inverse Probability Weighting Analysis: Association of Pet Ownership in Wave 5 With Cognitive Decline During Waves 5 to 9, Stratified by Living Alone in Wave 5 eTable 9. The Inverse Probability Weighting Analysis: Joint Associations of Pet Ownership and Living Alone in Wave 5 With Cognitive Decline During Waves 5 to 9 eTable 10. Association of Time-Varying Living Alone With Cognitive Decline During Waves 5 to 9 eFigure 1. The Timeline and Cumulative Attrition Rates of This Study eFigure 2. The Inclusion and Exclusion Process of Participants in This Study eFigure 3. Cumulative Rates of Loss to Follow-Up During Waves 5 to 9 eFigure 4. Actual Verbal Memory and Verbal Fluency Scores During Waves 5 to 9 eFigure 5. Estimated Cognition z Scores (SD Units) During Waves 5 to 9 by Pet Ownership in Wave 5, Stratified by Living Alone in Wave 5 eFigure 6. Estimated Cognition z Scores (SD Units) During Waves 5 to 9 by the Combination of Pet Ownership and Living Alone in Wave 5 eReferences [file jamanetwopen-e2349241-s001.pdf]

Supplementary Online Content

Li Y, Wang W, Zhu L, et al. Pet ownership, living alone, and cognitive decline among adults 50 years and older. *JAMA Netw Open*. 2023;6(12):e2349241. doi:10.1001/jamanetworkopen.2023.49241

eMethods. Assessment of Cognitive Function and Potential Covariates and Inverse Probability Weighting
eTable 1. Baseline Characteristics of Participants Included in the Analysis and Participants Lost to Follow-Up
eTable 2. Follow-Up Time of Participants During Waves 5 to 9
eTable 3. Rates of Loss to Follow-Up During Waves 5 to 9
eTable 4. Modifying Role of Living Alone in Wave 5 in the Association of Pet Ownership in Wave 5 With Cognitive Decline During Waves 5 to 9
eTable 5. The Inverse Probability Weighting Analysis: Association of Pet Ownership in Wave 5 With Cognitive Decline During Waves 5 to 9
eTable 6. The Inverse Probability Weighting Analysis: Association of Living Alone in Wave 5 With Cognitive Decline During Waves 5 to 9
eTable 7. The Inverse Probability Weighting Analysis: Modifying Role of Living Alone in Wave 5 in the Association of Pet Ownership in Wave 5 With Cognitive Decline During Waves 5 to 9
eTable 8. The Inverse Probability Weighting Analysis: Association of Pet Ownership in Wave 5 With Cognitive Decline During Waves 5 to 9, Stratified by Living Alone in Wave 5
eTable 9. The Inverse Probability Weighting Analysis: Joint Associations of Pet Ownership and Living Alone in Wave 5 With Cognitive Decline During Waves 5 to 9
eTable 10. Association of Time-Varying Living Alone With Cognitive Decline During Waves 5 to 9
eFigure 1. The Timeline and Cumulative Attrition Rates of This Study
eFigure 2. The Inclusion and Exclusion Process of Participants in This Study
eFigure 3. Cumulative Rates of Loss to Follow-Up During Waves 5 to 9
eFigure 4. Actual Verbal Memory and Verbal Fluency Scores During Waves 5 to 9
eFigure 5. Estimated Cognition *z* Scores (SD Units) During Waves 5 to 9 by Pet Ownership in Wave 5, Stratified by Living Alone in Wave 5
eFigure 6. Estimated Cognition *z* Scores (SD Units) During Waves 5 to 9 by the Combination of Pet Ownership and Living Alone in Wave 5
eReferences

This supplementary material has been provided by the authors to give readers additional information about their work.

© 2023 Li Y et al. *JAMA Network Open*.

eMethods. Assessment of Cognitive Function and Potential Covariates and Inverse Probability Weighting

Assessment of Cognitive Function

Verbal memory was assessed by testing the immediate and delayed recall of 10 unrelated words. The verbal memory score was the sum of words correctly recalled in the immediate and delayed word recall tests, ranging from 0 to 20. Verbal fluency was evaluated using a verbal fluency task in which participants were required to list as many animal names as possible within 1 min. The number of listed animal names was counted as the verbal fluency score, without upper limits. For both tests, a higher score suggests better cognitive performance. In addition, the cognitive assessment date of each participant was recorded.

Assessments of Potential Covariates

Race/ethnicity was self-reported and was categorized as White or other (including Asian, Asian British, Black, Black British, mixed ethnic group, and other).[1] Participants self-reported the highest educational qualification obtained. Education level was classified into high level (university degree or equivalent), middle level (A-level/higher education below degree), and low level (no qualifications/O-level or equivalent).[2] Wealth was based on a comprehensive assessment of economic resources (e.g., financial, housing, and physical wealth) excluding pension wealth, and was divided into quintiles (1 = poorest; 5 = richest).[3] Social isolation was evaluated using the previously reported Social Isolation Index.[4,5] This index included marital status (1 point for unmarried/not cohabiting), contacts with children (1

© 2023 Li Y et al. *JAMA Network Open*.

point for contacting in person or by telephone/written/e-mail less than monthly), contacts with other family members (1 point for contacting in person or by telephone/written/e-mail less than monthly), contacts with friends (1 point for contacting in person or by telephone/written/e-mail less than monthly), and social participation (1 point for not being a member of any organizations such as religious groups, gyms/sports clubs, and committees). The social isolation score ranges from 0 to 5, with a higher score representing more severe social isolation. According to the frequency of drinking in the past year, we classified drinking status into less than weekly, 1−4 days a week, and 5−7 days a week.[3] Physical activity was assessed by asking participants how often they participated in vigorous, moderate, and light physical activities, and was categorized into vigorous, moderate, and light physical activities.[6] Depressive symptoms were assessed using the 8-item version of the Center for Epidemiologic Studies Depression Scale. The total score ranges from 0 to 8, and a higher score indicates more severe depressive symptoms. A cut-off value of ≥ 4 was used to identify older adults with depressive symptoms.[7]

Inverse Probability Weighting

Referring to previous studies,[8,9] weights were derived from a logistic regression analysis between a set of measures that were independently predictive of missing data (i.e., age, sex, race/ethnicity, education level, employment status, wealth, smoking status, drinking status, physical activity, and the social isolation score, self-rated general health, depressive symptoms, hypertension, diabetes, and cardiovascular disease). We predict the probability

© 2023 Li Y et al. *JAMA Network Open*.

that each individual is a complete case (i.e., non-missing) using logistic regression. The inverse of the above probabilities is the weight. Minimal missing data on indicators used to derive weights were singly imputed as the modal value of continuous variables or mean value of categorical variables (all indicators had < 6% of values missing). The Hosmer-Lemeshow test was used assess the fit of the missingness model, with results showing no indication of poor fit (*P*-value < 0.05). Weights ranged from 1.01 to 7.03. The inverse probability weighting analysis is performed by adding weights in the main analysis.

© 2023 Li Y et al. *JAMA Network Open*.

eTable 1. Baseline Characteristics of Participants Included in the Analysis and Participants Lost to Follow-Up

**Characteristic**	**Participants included**	**Participants lost to follow-up**	***P*-value**
No.	7945	691	
Age, years, mean (SD)	66.3 (8.8)	67.4 (10.5)	.13
Male, No. (%)	3499 (44.0)	328 (47.5)	.08
White race, No. (%)	7746 (97.5)	675 (97.7)	.76
Education level, No. (%)			.22
High	2549 (32.1)	200 (28.9)	
Middle	638 (8.0)	55 (8.0)	
Low	4758 (59.9)	436 (63.1)	
Employment status, No. (%)			.07
Employed	2553 (32.1)	193 (27.9)	
Unemployed	862 (10.9)	82 (11.9)	
Retired	4530 (57.0)	416 (60.2)	
Wealth quintiles, No. (%)			.07
1 (Poorest)	1117 (14.1)	119 (17.2)	
2	1423 (17.9)	125 (18.1)	
3	1421 (17.9)	132 (19.1)	
4	1528 (19.2)	111 (16.1)	
5 (Richest)	2456 (30.9)	204 (29.5)	
Living alone, No. (%)	2139 (26.9)	199 (28.8)	.29
The social isolation score, mean (SD)	0.87 (0.88)	0.89 (0.89)	.32
Current smokers, No. (%)	961 (12.1)	98 (14.2)	.11
Drinking status, No. (%)			.09
Less than weekly	3139 (39.5)	295 (42.7	
1−4 days a week	2926 (36.8)	220 (31.8)	
5−7 days a week	1880 (23.7)	176 (25.5)	
Physical activity, No. (%)			.15
Light	1614 (18.6)	140 (20.3)	
Moderate	3543 (44.6)	288 (41.7)	
Vigorous	2788 (36.8)	263 (38.1)	
Self-rated general health, No. (%)			.19
Excellent	1030 (13.0)	81 (11.7)	
Very good	2482 (31.2)	202 (29.2)	
Good	2557 (32.2)	217 (31.45)	
Fair	1392 (17.5)	140 (20.3)	
Poor	484 (6.1)	51 (7.4)	
Depressive symptoms, No. (%)	930 (11.7)	95 (13.8)	.11
Hypertension, No. (%)	3133 (39.4)	348 (50.4)	<.001

© 2023 Li Y et al. *JAMA Network Open*.

Diabetes, No. (%)	825 (10.4)	81 (11.7)	.27
Cardiovascular disease, No. (%)	1726 (21.7)	219 (31.7)	<.001
The actual verbal memory score, mean (SD)	10.8 (3.5)	10.5 (3.7)	.10
The actual verbal fluency score, mean (SD)	21.4 (6.6)	21.3 (6.5)	.82

Two independent sample *t*-tests were used to compare the means of continuous variables.

Pearson Chi-squared tests were performed to compare the distribution of categorical variables.

Abbreviation: SD, standard deviation.

© 2023 Li Y et al. *JAMA Network Open*.

eTable 2. Follow-Up Time of Participants During Waves 5 to 9

	**Follow-up time, years**				***P*-value**
	**Median**	**P_{25} to P_{75}**	**Min**	**Max**	
**Total**	7.9	5.9 to 8.1	1.3	9.0	
**Pet ownership** [a]					.10
Yes	7.9	5.8 to 8.1	1.3	9.0	
No	7.9	6.0 to 8.1	1.6	9.0	
**Living alone** [a]					.12
Yes	7.9	6.0 to 8.1	1.3	9.0	
No	7.8	4.1 to 8.1	1.5	9.0	
**Pet ownership and living alone** [b]					.07
Pet ownership (yes) and living alone (no)	8.0	6.0 to 8.1	1.6	8.9	
Pet ownership (no) and living alone (no)	7.9	5.9 to 8.1	1.3	9.0	
Pet ownership (yes) and living alone (yes)	7.9	5.8 to 8.1	1.6	9.0	
Pet ownership (no) and living alone (yes)	7.8	4.0 to 8.1	1.5	8.9	

[a] Wilcoxon rank-sum tests were used to compare the distribution of follow-up time.

[b] Kruskal-Wallis rank tests were performed to compare the distribution of follow-up time.

© 2023 Li Y et al. *JAMA Network Open*.

eTable 3. Rates of Loss to Follow-Up During Waves 5 to 9 [a]

	Total	Loss to follow-up	Rate of loss to follow-up, %	*P*-value
**Total**	8636	691	8.0	
**Pet ownership**				.17
Yes	3016	225	8.3	
No	5620	466	7.5	
**Living alone**				.08
Yes	2387	211	8.8	
No	6249	480	7.7	
**Pet ownership and living alone**				.06
Pet ownership (yes) and living alone (no)	2327	159	6.8	
Pet ownership (no) and living alone (no)	3922	321	8.2	
Pet ownership (yes) and living alone (yes)	689	66	9.6	
Pet ownership (no) and living alone (yes)	1698	145	8.5	

[a] Pearson Chi-squared tests were performed to compare the distribution of rates of loss to follow-up.

© 2023 Li Y et al. *JAMA Network Open*.

eTable 4. Modifying Role of Living Alone in Wave 5 in the Association of Pet Ownership in Wave 5 With Cognitive Decline During Waves 5 to 9 [a]

	β **coefficient (95% CI)**	***P*-value**
**Composite verbal cognition**		
Pet ownership (no) × living alone (no) × time	0 [Reference]	
Pet ownership (yes) × living alone (yes) × time	0.021 (0.007 to 0.035)	.003
**Verbal memory**		
Pet ownership (no) × living alone (no) × time	0 [Reference]	
Pet ownership (yes) × living alone (yes) × time	0.020 (0.006 to 0.035)	.005
**Verbal fluency**		
Pet ownership (no) × living alone (no) × time	0 [Reference]	
Pet ownership (yes) × living alone (yes) × time	0.015 (0.001 to 0.030)	.04

[a] The model included pet ownership, living alone, time, pet ownership × time, living alone × time, pet ownership × living alone, pet ownership × living alone × time, and covariates (i.e., age, sex, race/ethnicity, education level, employment status, wealth, smoking status, drinking status, physical activity, the social isolation score, self-rated general health, depressive symptoms, hypertension, diabetes, and cardiovascular disease in wave 5).

Abbreviation: CI, confidence interval.

© 2023 Li Y et al. *JAMA Network Open*.

eTable 5. The Inverse Probability Weighting Analysis: Association of Pet Ownership in Wave 5 With Cognitive Decline During Waves 5 to 9 [a]

	β **coefficient (95% CI)**	***P*-value**
**Composite verbal cognition**		
Pet ownership (no) × time	0 [Reference]	
Pet ownership (yes) × time	0.008 (0.002 to 0.014)	.01
**Verbal memory**		
Pet ownership (no) × time	0 [Reference]	
Pet ownership (yes) × time	0.007 (0.001 to 0.013)	.03
**Verbal fluency**		
Pet ownership (no) × time	0 [Reference]	
Pet ownership (yes) × time	0.007 (0.000 to 0.014)	.04

[a] The model included pet ownership, time, pet ownership × time, and covariates (i.e., age, sex, race/ethnicity, education level, employment status, wealth, smoking status, drinking status, physical activity, the social isolation score, self-rated general health, depressive symptoms, hypertension, diabetes, cardiovascular disease, and living alone in wave 5).

Abbreviation: CI, confidence interval.

© 2023 Li Y et al. *JAMA Network Open*.

eTable 6. The Inverse Probability Weighting Analysis: Association of Living Alone in Wave 5 With Cognitive Decline During Waves 5 to 9 [a]

	**β coefficient (95% CI)**	***P*-value**
**Composite verbal cognition**		
Living alone (no) × time	0 [Reference]	
Living alone (yes) × time	-0.021 (-0.028 to -0.015)	<.001
**Verbal memory**		
Living alone (no) × time	0 [Reference]	
Living alone (yes) × time	-0.019 (-0.026 to -0.013)	<.001
**Verbal fluency**		
Living alone (no) × time	0 [Reference]	
Living alone (yes) × time	-0.015 (-0.022 to -0.008)	<.001

[a] The model included living alone, time, living alone × time, and covariates (i.e., age, sex, race/ethnicity, education level, employment status, wealth, smoking status, drinking status, physical activity, the social isolation score, self-rated general health, depressive symptoms, hypertension, diabetes, cardiovascular disease, and pet ownership in wave 5).

Abbreviation: CI, confidence interval.

© 2023 Li Y et al. *JAMA Network Open*.

eTable 7. The Inverse Probability Weighting Analysis: Modifying Role of Living Alone in Wave 5 in the Association of Pet Ownership in Wave 5 With Cognitive Decline During Waves 5 to 9 [a]

	***β* coefficient (95% CI)**	***P*-value**
**Composite verbal cognition**		
Pet ownership (no) × living alone (no) × time	0 [Reference]	
Pet ownership (yes) × living alone (yes) × time	0.021 (0.006 to 0.035)	.005
**Verbal memory**		
Pet ownership (no) × living alone (no) × time	0 [Reference]	
Pet ownership (yes) × living alone (yes) × time	0.020 (0.005 to 0.035)	.009
**Verbal fluency**		
Pet ownership (no) × living alone (no) × time	0 [Reference]	
Pet ownership (yes) × living alone (yes) × time	0.015 (0.000 to 0.030)	.049

[a] The model included pet ownership, living alone, time, pet ownership × time, living alone × time, pet ownership × living alone, pet ownership × living alone × time, and covariates (i.e., age, sex, race/ethnicity, education level, employment status, wealth, smoking status, drinking status, physical activity, the social isolation score, self-rated general health, depressive symptoms, hypertension, diabetes, and cardiovascular disease in wave 5).

Abbreviation: CI, confidence interval.

© 2023 Li Y et al. *JAMA Network Open*.

eTable 8. The Inverse Probability Weighting Analysis: Association of Pet Ownership in Wave 5 With Cognitive Decline During Waves 5 to 9, Stratified by Living Alone in Wave 5

	***β* coefficient (95% CI)**	***P*-value**
**Living alone (yes)** [a]		
**Composite verbal cognition**		
Pet ownership (no) × time	0 [Reference]	
Pet ownership (yes) × time	0.023 (0.010 to 0.035)	<.001
**Verbal memory**		
Pet ownership (no) × time	0 [Reference]	
Pet ownership (yes) × time	0.021 (0.008 to 0.034)	.002
**Verbal fluency**		
Pet ownership (no) × time	0 [Reference]	
Pet ownership (yes) × time	0.017 (0.004 to 0.031)	.01
**Living alone (no)** [b]		
**Composite verbal cognition**		
Pet ownership (no) × time	0 [Reference]	
Pet ownership (yes) × time	0.002 (-0.005 to 0.009)	.61
**Verbal memory**		
Pet ownership (no) × time	0 [Reference]	
Pet ownership (yes) × time	0.001 (-0.006 to 0.008)	.84
**Verbal fluency**		
Pet ownership (no) × time	0 [Reference]	
Pet ownership (yes) × time	0.003 (-0.005 to 0.010)	.50

[a] The model included pet ownership, time, pet ownership × time, and covariates (i.e., age, sex, race/ethnicity, education level, employment status, wealth, smoking status, drinking status, physical activity, the social isolation score, self-rated general health, depressive symptoms, hypertension, diabetes, cardiovascular disease, and living alone in wave 5).

[b] The model included living alone, time, living alone × time, and covariates (i.e., age, sex, race/ethnicity, education level, employment status, wealth, smoking status, drinking status, physical activity, the social isolation score, self-rated general health, depressive symptoms, hypertension, diabetes, cardiovascular disease, and pet ownership in wave 5).

Abbreviation: CI, confidence interval.

eTable 9. The Inverse Probability Weighting Analysis: Joint Associations of Pet Ownership and Living Alone in Wave 5 With Cognitive Decline During Waves 5 to 9 [a]

	***β* coefficient (95% CI)**	***P*-value**
**Composite verbal cognition**		
Living alone (no) and pet ownership (yes) × time	0 [Reference]	
Living alone (no) and pet ownership (no) × time	-0.002 (-0.009 to 0.005)	.61
Living alone (yes) and pet ownership (yes) × time	-0.007 (-0.019 to 0.005)	.27
Living alone (yes) and pet ownership (no) × time	-0.029 (-0.038 to -0.020)	<.001
**Verbal memory**		
Living alone (no) and pet ownership (yes) × time	0 [Reference]	
Living alone (no) and pet ownership (no) × time	-0.001 (-0.008 to 0.006)	.82
Living alone (yes) and pet ownership (yes) × time	-0.006 (-0.018 to 0.007)	.37
Living alone (yes) and pet ownership (no) × time	-0.026 (-0.035 to -0.017)	<.001
**Verbal fluency**		
Living alone (no) and pet ownership (yes) × time	0 [Reference]	
Living alone (no) and pet ownership (no) × time	-0.003 (-0.010 to 0.005)	.50
Living alone (yes) and pet ownership (yes) × time	-0.005 (-0.017 to 0.008)	.47
Living alone (yes) and pet ownership (no) × time	-0.022 (-0.032 to -0.013)	<.001

[a] The model included the combination of living alone and pet ownership, time, the combination of living alone and pet ownership × time, and covariates (i.e., age, sex, race/ethnicity, education level, employment status, wealth, smoking status, drinking status, physical activity, the social isolation score, self-rated general health, depressive symptoms, hypertension, diabetes, and cardiovascular disease in wave 5).

Abbreviation: CI, confidence interval.

© 2023 Li Y et al. *JAMA Network Open*.

eTable 10. Association of Time-Varying Living Alone With Cognitive Decline During Waves 5 to 9 [a]

	***β* coefficient (95% CI)**	***P*-value**
**Composite verbal cognition**		
Pet ownership (no) × time	0 [Reference]	
Pet ownership (yes) × time	-0.022 (-0.028 to -0.015)	<.001
**Verbal memory**		
Pet ownership (no) × time	0 [Reference]	
Pet ownership (yes) × time	-0.020 (-0.026 to -0.015)	<.001
**Verbal fluency**		
Pet ownership (no) × time	0 [Reference]	
Pet ownership (yes) × time	-0.018 (-0.024 to -0.010)	<.001

[a] The model included living alone, time, living alone × time, and covariates (i.e., age, sex, race/ethnicity, education level, employment status, wealth, smoking status, drinking status, physical activity, the social isolation score, self-rated general health, depressive symptoms, hypertension, diabetes, cardiovascular disease, and pet ownership in wave 5).

Abbreviation: CI, confidence interval.

© 2023 Li Y et al. *JAMA Network Open*.

eFigure 1. The Timeline and Cumulative Attrition Rates of This Study

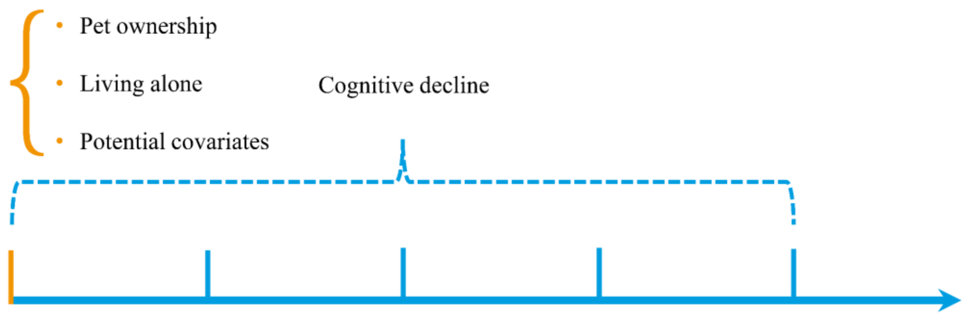

**Wave**	Wave 5 (baseline)	Wave 6	Wave 7	Wave 8	Wave 9
**Year**	2010–2011	2012–2013	2014–2015	2016–2017	2018–2019
**No.**	7945	7786	6780	6035	5345
**Cumulative attrition rate**	0.0%	2.0%	14.7%	24.0%	32.7%

© 2023 Li Y et al. *JAMA Network Open*.

eFigure 2. The Inclusion and Exclusion Process of Participants in This Study

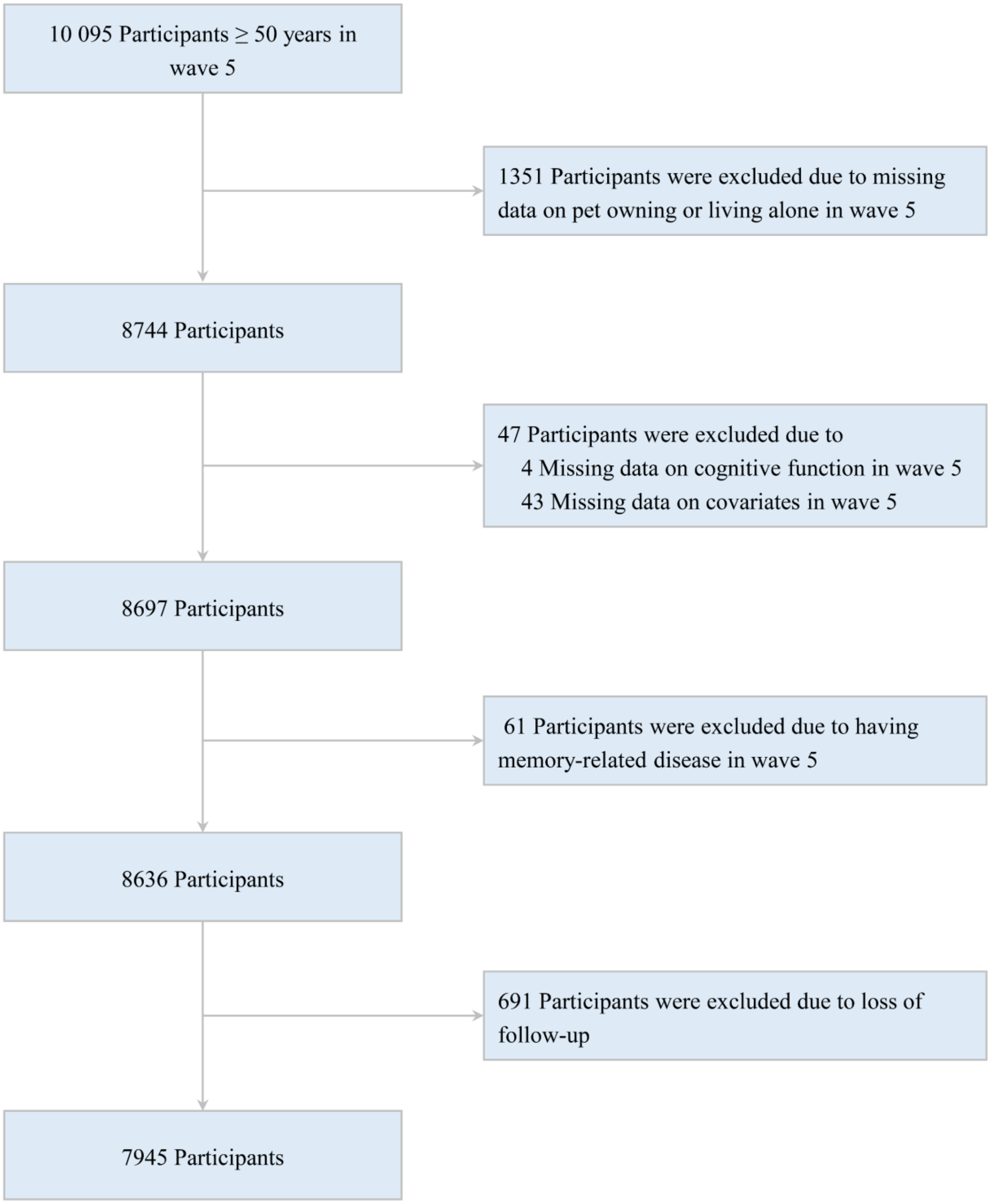

© 2023 Li Y et al. *JAMA Network Open*.

eFigure 3. Cumulative Rates of Loss to Follow-Up During Waves 5 to 9

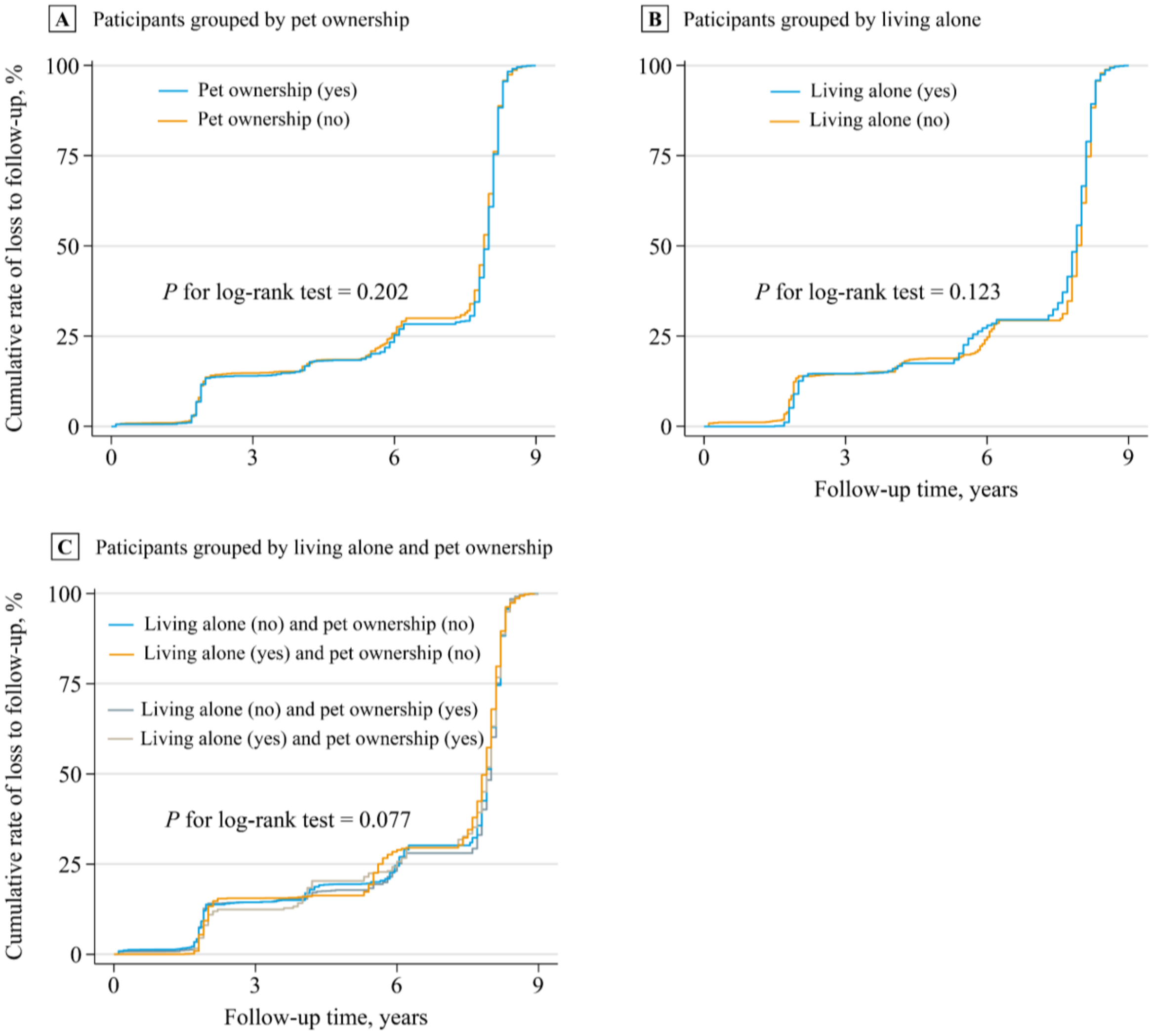

© 2023 Li Y et al. *JAMA Network Open*.

eFigure 4. Actual Verbal Memory and Verbal Fluency Scores During Waves 5 to 9

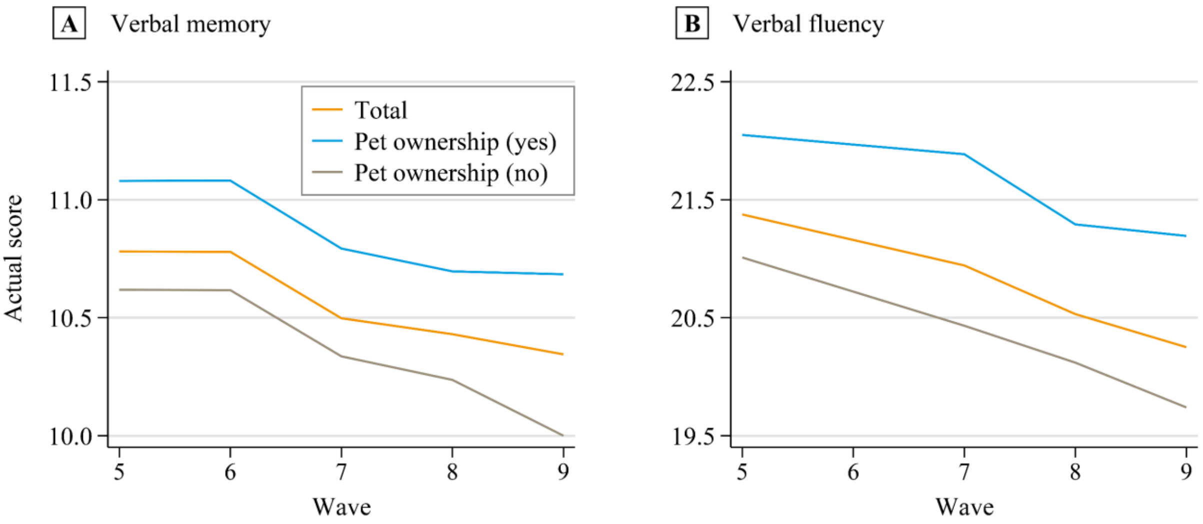

© 2023 Li Y et al. *JAMA Network Open*.

eFigure 5. Estimated Cognition *z* Scores (SD Units) During Waves 5 to 9 by Pet Ownership in Wave 5, Stratified by Living Alone in Wave 5

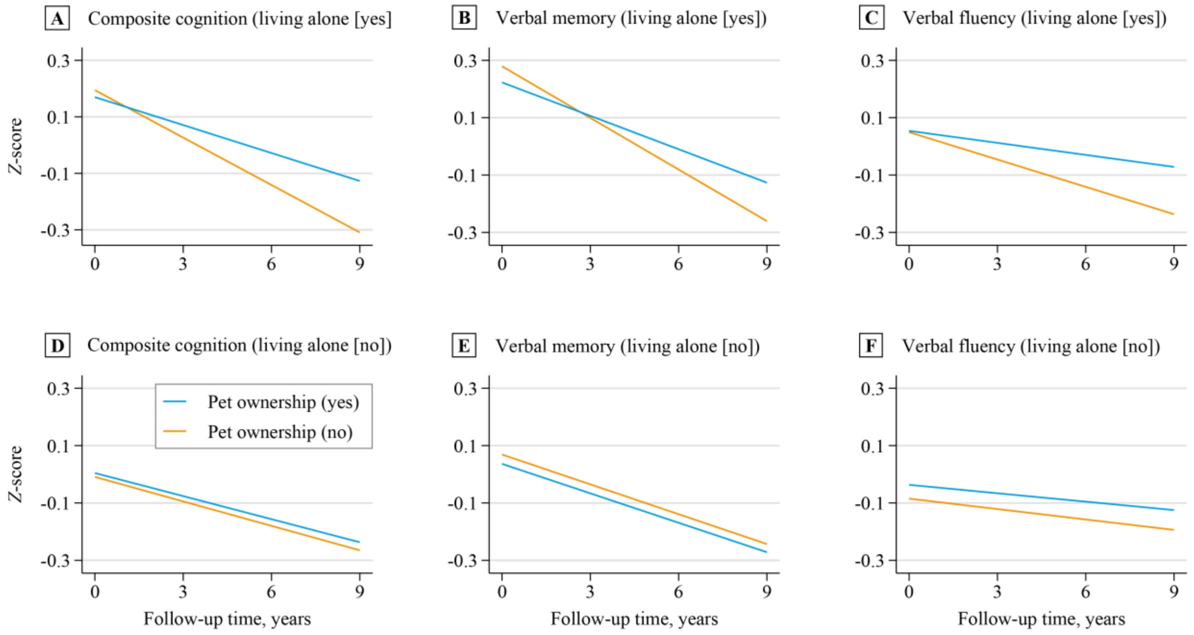

Covariates were set to the following values: age = 65 years, female, White race, high education level, retired, the third quintile of wealth, the social isolation score = 1, current non-smokers, drinking less than once a week, moderate physical activity, good self-rated general health, and not having depressive symptoms, hypertension, diabetes, and cardiovascular disease.

© 2023 Li Y et al. *JAMA Network Open*.

eFigure 6. Estimated Cognition *z* Scores (SD Units) During Waves 5 to 9 by the Combination of Pet Ownership and Living Alone in Wave 5

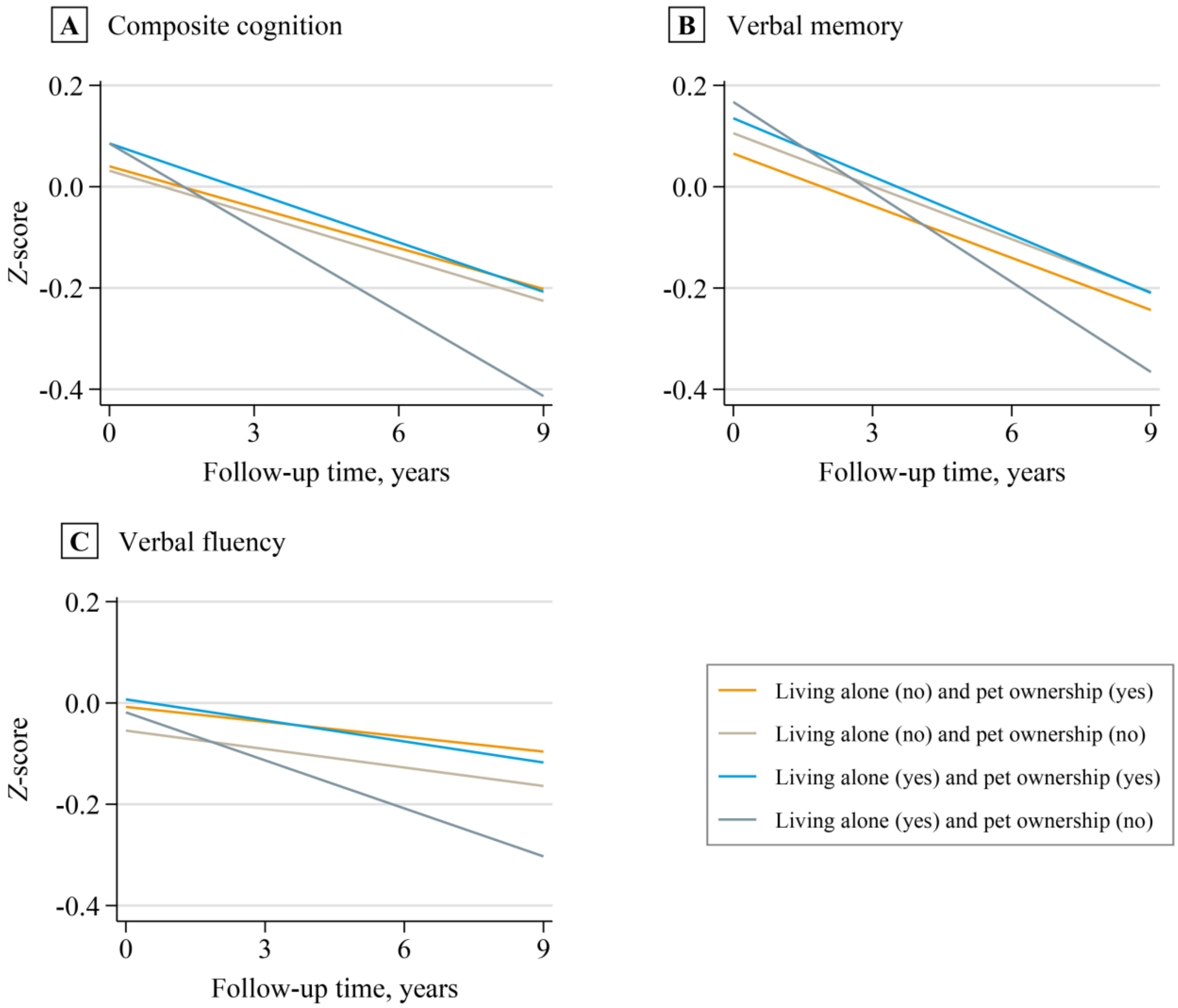

Covariates were set to the following values: age = 65 years, female, white race, high education level, retired, the third quintile of wealth, the social isolation score = 1, current non-smokers, drinking less than once a week, moderate physical activity, good self-rated general health, and not having depressive symptoms, hypertension, diabetes, and cardiovascular disease.

© 2023 Li Y et al. *JAMA Network Open*.

eReferences

1. Yu X, Langa KM, Cho TC, Kobayashi LC. Association of Perceived Job Insecurity With Subsequent Memory Function and Decline Among Adults 55 Years or Older in England and the US, 2006 to 2016. *JAMA Netw Open*. 2022;5(4):e227060. doi:10.1001/jamanetworkopen.2022.7060
2. Li Y, Cheng L, Guo L, et al. Mediating role of personality traits in the association between multi-dimensional adverse childhood experiences and depressive symptoms among older adults: A 9-year prospective cohort study. *J Affect Disord*. 2023;331:167-174. doi:10.1016/j.jad.2023.03.067
3. Li Y, Wang X, Guo L, et al. Eight-Year Trajectories of Late-Life Loneliness and Incident Dementia: A Nationally Representative Cohort Study. *Am J Geriatr Psychiatry*. 2022;31(7):475-486. doi:10.1016/j.jagp.2022.12.002
4. Maharani A, Pendleton N, Leroi I. Hearing Impairment, Loneliness, Social Isolation, and Cognitive Function: Longitudinal Analysis Using English Longitudinal Study on Ageing. *Am J Geriatr Psychiatry*. 2019;27(12):1348-1356. doi:10.1016/j.jagp.2019.07.010
5. Smith TO, Dainty JR, MacGregor A. Trajectory of social isolation following hip fracture: an analysis of the English Longitudinal Study of Ageing (ELSA) cohort. *Age Ageing*. 2018;47(1):107-112. doi:10.1093/ageing/afx129
6. Iob E, Lacey R, Steptoe A. The long-term association of adverse childhood experiences with C-reactive protein and hair cortisol: Cumulative risk versus dimensions of adversity. *Brain Behav Immun*. 2020;87:318-328. doi:10.1016/j.bbi.2019.12.019
7. Li C, Zhu Y, Ma Y, Hua R, Zhong B, Xie W. Association of Cumulative Blood Pressure With Cognitive Decline, Dementia, and Mortality. *J Am Coll Cardiol*. 2022;79(14):1321-1335. doi:10.1016/j.jacc.2022.01.045
8. Seaman SR, White IR. Review of inverse probability weighting for dealing with missing data. *Stat Methods Med Res*. 2013;22(3):278-295. doi:10.1177/0962280210395740
9. Hammerton G, Lewis G, Heron J, Fernandes G, Hickman M, Lewis G. The association of alcohol dependence and consumption during adolescence with depression in young adulthood, in England: a prospective cohort study. *Lancet Psychiatry*. 2023;10(7):490-498. doi:10.1016/s2215-0366(23)00138-4

© 2023 Li Y et al. *JAMA Network Open*.
