# Supplementary material for: Pet Ownership, Living Alone, and Cognitive Decline Among Adults 50 Years and Older
Source: JAMA Netw Open. 2023 Dec 26;6(12):e2349241. doi: 10.1001/jamanetworkopen.2023.49241 (PMC10751597; doi:10.1001/jamanetworkopen.2023.49241)
Supplement: Supplement 2. — Data Sharing Statement [file jamanetwopen-e2349241-s002.pdf]

Data Sharing Statement

Li. Pet Ownership, Living Alone, and Cognitive Decline Among Adults 50 Years and Older. *JAMA Netw Open*. Published December 26, 2023. doi:10.1001/jamanetworkopen.2023.49241

Data
Data available: Yes
Data types: Participant data with identifiers
How to access data: The ELSA datasets were available from the UK Data Service (https://ukdataservice.ac.uk/).
When available: With publication

Supporting Documents
Document types: None

Additional Information
Who can access the data: researchers whose proposed use of the data has been approved
Types of analyses: A specified purpose
Mechanisms of data availability: After approval of a proposal
